# Retention of adults from fishing communities in an HIV vaccine preparedness study in Masaka, Uganda

**DOI:** 10.1371/journal.pone.0198460

**Published:** 2019-01-14

**Authors:** Ubaldo Mushabe Bahemuka, Andrew Abaasa, Eugene Ruzagira, Christina Lindan, Matt A. Price, Anatoli Kamali, Pat Fast

**Affiliations:** 1 Medical Research Council (MRC)/Uganda Virus Research Institute (UVRI) and London School of Hygiene and Tropical Medicine Uganda Research Unit, Entebbe, Uganda; 2 University of California, San Francisco, United States of America; 3 International AIDS Vaccine Initiative, New York, United States of America; Beth Israel Deaconess Medical Center/Harvard Medical School, UNITED STATES

## Abstract

**Introduction:**

People living in fishing communities around Lake Victoria may be suitable for enrolment in HIV prevention trials because of high HIV incidence. We assessed the ability to recruit and retain individuals from fishing communities into an HIV vaccine preparedness cohort study in Masaka, Uganda.

**Methods:**

HIV high risk, sero-negative adults (18–49 years) were identified from four fishing villages bordering Lake Victoria through door-to-door HIV counselling and testing (HCT). Interested persons were referred for screening, enrolment, and quarterly follow-up visits at a study clinic located approximately 30–40 kilometres away. Repeat HCT, HIV risk assessment, and evaluation and treatment for sexually transmitted infections were provided. Rates of and factors associated with study dropout were assessed using Poisson regression models.

**Results:**

A total of 940 participants were screened between January 2012 and February 2015, of whom 654 were considered for the analysis. Over a two-year follow-up period, 197 (30.1%) participants dropped out of the study over 778.9 person-years, a dropout rate of 25.3 / 100 person-years of observation. Dropout was associated with being female (aRR = 1.56, 95% confidence interval [CI] 1.12–2.18), being 18–24 years (aRR = 1.64; 95% CI 1.03–2.60) or being 25–34 years (aRR = 1.63; 95% CI 1.04–2.55) compared to being 35+ years; having no education (aRR = 2.02; 95% CI: 1.23–3.31); living in the community for less than one year (aRR = 2.22; 95% CI: 1.46–3.38), or 1–5 years (aRR = 1.68; 95% CI: 1.16–2.45), compared to more than five years.

**Conclusions:**

Our results suggest that individuals from fishing communities can be recruited and retained in longitudinal studies; however, intensified participant tracing may be necessary for women, younger volunteers, those who are less educated and new residents.

## Introduction

New HIV infections continue to occur in sub-Saharan Africa, despite behavioural and biomedical prevention efforts[1]. Therefore, development of an effective HIV vaccine will be essential to reducing incidence in this region[2]. Efficacy testing of HIV vaccine candidates is more efficient among populations with favourable retention and high HIV incidence rates[3]. Fishing communities in Uganda have a high burden of HIV with HIV prevalence and incidence ranging from 20–40 percent [4–6] and 3–9 cases per 100 person-years of observation (PYO), respectively [7–9]. This high level of risk suggests that people living in these communities may be ideal participants for HIV vaccine efficacy trials. However, fishing communities are typically located in remote areas and often have little if any health care infrastructure, factors that may present challenges to the rigorous implementation requirements of HIV vaccine preparedness and efficacy studies[10].

A number of studies have evaluated the suitability and retention of members of fishing communities around Lake Victoria in vaccine preparedness trials. In three recent studies, the 12-month retention was reported to range between 77% to 85% [9, 11, 12]. Recruitment and follow up assessments for these studies, however, were conducted within the fishing villages themselves. During the conduct of a Simulated HIV vaccine trial(SiVET), 73% 12-month retention was reported in the observational arm [13].

Because evaluation of vaccine efficacy trials requires sophisticated clinical and laboratory facilities for diagnostics and sample processing and storage, it may not be feasible to place these facilities within remote communities from where participants are recruited. Little is known about whether members of fishing communities would participate in a study if they were required to travel for study visits. Therefore, we assessed the acceptability of using a research clinic that was situated in an urban center located 30–40 kilometers from four selected fishing communities, and evaluated retention in a vaccine preparedness study.

## Materials and methods

### Study setting, participant identification and recruitment

The International AIDS Vaccine Initiative (IAVI) in collaboration with the Medical Research Council/Uganda Virus Research Institute Research (MRC/UVRI) has supported a clinical research centre in rural southwestern Uganda with the aim of conducting future HIV vaccine efficacy trials. The research centre is located in Masaka town, approximately 30–40 kilometers (km) away from the shores of Lake Victoria. The clinic equipped to conduct HIV vaccine trials, has a Good Clinical Laboratory Practice (GCLP)-accredited laboratory, a vaccine pharmacy, and Good Clinical Practice (GCP)-trained staff [14]. The HIV vaccine preparedness study described here was conducted among individuals identified from four mainland fishing communities on the shores of Lake Victoria (called landing-sites) located in Masaka and Kalungu districts. These communities were selected because they were the most populous fishing communities (with ≥1000 adult residents) in these two districts.

Since January 2012 the study field team consisting of both male and female counselors visited houses, docked boats, fishing stalls and other venues at the four landing-sites, to offer free on-site rapid HIV testing and counseling for adults daily. Persons aged 18–49 years old who were identified as being HIV uninfected were asked if they would be willing to enroll into a longitudinal study that would require repeated travel to a research clinic. Those who had a positive rapid test result underwent confirmatory HIV testing by having venous blood samples re-tested at the MRC/UVRI laboratory in Entebbe. Those with confirmed HIV positive results were asked to come to the research clinic to receive their results. HIV-infected persons were referred for care at a treatment center of their choice.

HIV-uninfected individuals interested in the vaccine preparedness study and who travelled to the research clinic were given detailed information about study procedures in a group setting. Individuals also had one-on-one sessions with a study nurse-counselor who provided additional study information, answered questions, and obtained written informed consent. After signing consent, participants were assessed for eligibility based on the following criteria: being 18–49 years, HIV uninfected, and sexually active (defined as having had sex at least once in the last the three months). Participants also needed to be considered at high risk of HIV acquisition by reporting at least one of the following: self-reported history of a sexually transmitted infection (STI) in the last three months, or the presence of an STI based on medical history/physical exam; condomless vaginal or anal intercourse with a new or with more than one sexual partner in the past three months; staying away from home for at least two nights in the past three months; drinking alcohol at least once a week or using illicit drugs (marijuana, Khat, or any other stimulants) in the past month. Participants who fulfilled the above criteria were enrolled into the study and completed an interviewer-administered questionnaire on socio-demographics in either the local language (Luganda) or English. The study physician obtained a medical history and performed a physical exam to assess for the presence of STIs and circumcision status among males. Participants provided their physical address and phone numbers to facilitate future contact and follow-up.

### Follow-up visits and study procedures

Participants returned to the clinic every three months for repeat HIV counselling and testing, provision of updated locator information and interim medical history. Everyone underwent a symptom-directed physical exam and a clinical evaluation for STIs. STI treatment was provided as per National Guidelines. Every six months’ participants provided information on HIV risk behaviour. At the 12-month visit, they were evaluated to determine if they continued to fulfil the eligibility requirements for participation in the study based on being at high risk of HIV, according to the criteria described above. Participants who were no longer eligible were withdrawn from continuing in the study, but were not considered to have dropped out.

To bolster retention, study staff kept in touch with an existing Community Advisory Board sharing study experiences at regular meetings. Staff also implemented various community activities, including meetings to improve awareness of research, and activities such as football matches. In addition, participants were given cards indicating the date of their next visit and were called by phone two days prior to their visit to remind them to come to clinic. Ten study participants who were community peer leaders assisted with tracing those who had missed a visit by going to participants’ homes up to three times. Participants who missed two consecutive visits and could not be located, and/or refused to return were considered to have dropped-out.

Reimbursement was provided for transportation and time (5000 Uganda shillings, approximately 1.4 USD) at the end of each visit.

Rapid HIV antibody testing was performed on venous blood samples using a single rapid test, Alere Determine (Alere Medical Company Ltd. Chiba, Japan). Specimens that were positive by rapid test were tested at Entebbe with two enzyme linked immunosorbent assay (ELISA) tests in parallel (Murex Biotech Limited, Dartford, United Kingdom, and Vironostika, BioMérieux boxtel, The Netherlands). Discrepant ELISA test results were resolved by testing with either Statpak (Chembio Diagnostic Systems Inc., USA) or Western Blot (Cambridge Biotech, USA). The results of HIV negative rapid tests were given to participants immediately. Participants with confirmed HIV positive results were asked to come to the research site to receive their results. Syphilis testing, done at enrolment and then annually, was performed using the rapid plasma reagin (RPR) test (Microvue, Becton Dickson, Maryland, USA). Samples with an RPR titre of > = 1:8 were also tested using the Treponema Pallidum Hemaglutination Assay (TPHA) (Biotech Laboratories, UK). Active syphilis infection was defined as having both a positive RPR titre of > = 1:8 and a positive TPHA result.

### Statistical analysis

Data were analysed in Stata 14.0 (Stata Corp, College Station, TX, USA). Participant baseline characteristics were summarised using proportions and means, and stratified by gender. Proportions and means were used to compare baseline characteristics of participants who dropped out of the study to those that completed their scheduled follow up visits. Dropout was defined as all-cause (including withdrawal by self or investigator, death, loss-to-follow-up or refusal to continue) over a maximum of 24 months of follow-up. Participants who became ineligible at the annual reassessment of eligibility (criteria described above) were not considered to have dropped out, but were censored at 12 months. Persons who HIV sero-converted were not considered to have dropped out, but their follow up was censored at the estimated date of HIV infection, considered to be the midpoint between the date of the last negative and the first positive HIV test result. Seventy-two participants who were enrolled but did not return for any follow up visit were considered to have contributed one-month of follow-up time; this was done because Poisson regression analysis cannot include zero-months of follow-up. We did not want to remove these 72 people form the analysis entirely, as that would have underestimated drop-out. The dropout rate was estimated as the number of participants who dropped out divided by the total person-years of observation (PYO). The PYO were calculated as the sum of the time from enrolment (baseline) to the date of the last clinic visit or date of censoring. Dropout rates were compared by demographic and clinical characteristics. Rate ratios (RR) and 95% confidence intervals (CI) were calculated for the association of factors with dropping out using a Poisson regression model. All factors for which univariate associations attained a significance of p < 0.1 on the log-likelihood test were included in an initial multivariable model. Factors were retained in the multivariable model if the p-value for inclusion using the log-likelihood test was ≤0.05. We also calculated a Kaplan- Meier estimate of dropout rate over time for the participants in the cohort. We further performed a sensitivity analysis excluding the 72 who never returned for any follow up from the regression analyses of factors associated with drop-out.

### Ethical considerations

The study was approved by the Research and Ethics Committee of the Uganda Virus Research Institute, and by the Uganda National Council of Science and Technology.

## Results

### Screening and enrolment

A total of 940 individuals were screened between January 2012 and February 2015, we include follow up data through February 2015; 286 were not enrolled (245 were at low risk for HIV, eight were HIV-infected, 26 were excluded for other reasons). We present data on 654 (69.6%) participants who were due for at least one follow-up visit at the time of the analysis. Only six volunteers were not yet due for first follow up.

### Baseline characteristics

The majority (61.4%) of the participants were male (Table 1). The mean age was 27.7 years (SD 6.9). Nearly half (48.8%) were married, most (71.3%) had attained primary education and were engaged in fishing or related occupations (51.1%). The majority of the sample (76.9%) was of Christian faith and of Baganda ethnicity (44.0%). Only a third (30.7%) had lived in the fishing community for more than five years and 45.8% had stayed away from home for at least two days in the last three months. A majority (60.0%) reported having two to three sexual partners in the last three months, and about three quarters (74.1%) reported having a new sexual partner in the same period; 63.5% reported condom use with this new partner. The majority of the participants (70.6%) were recruited from one of the four fishing communities.

**Table 1:** Baseline socio-demographic and behavioural characteristics of men and women enrolled in a longitudinal HIV vaccine preparedness study, Masaka, Uganda.

Variable	All		Male		Female		
	N = 654		N = 402		N = 252		P-value of gender difference
	n	(%)	n	(%)	n	(%)	
**Age (years)**							0.003
35+	248	38.0	132	32.8	116	46.0	
25–34	282	43.0	186	46.3	96	38.1	
18–24	124	19.0	84	20.9	40	15.9	
**Current marital status**							<0.001
Single	186	28.4	132	32.8	54	21.4	
Married	319	48.8	217	54.0	102	40.5	
Separated/Widowed/divorced	149	22.8	53	13.2	96	38.1	
**Education level**							0.033
More than primary	125	19.1	66	16.4	59	23.4	
Primary	466	71.3	301	74.9	165	65.5	
None	63	9.6	35	8.7	28	11.1	
**Occupation**							<0.001
Small scale business	49	7.5	32	8.0	17	6.7	
Fishing/fishing related^1^	334	51.1	280	69.6	54	21.4	
Services(Bar/lodge/restaurant/saloon)	67	10.2	21	5.2	46	18.3	
Other(Peasant farmer/House wife)	204	31.2	69	17.2	135	53.6	
**Religion**							0.729
Muslim	151	23.1	91	22.6	60	23.8	
Christian	503	76.9	311	77.4	192	76.2	
**Tribe/Ethnic group**							<0.001
Baganda	288	44.0	178	44.3	110	43.7	
Banyankole	93	14.2	58	14.4	35	13.9	
Banyarwanda	136	20.8	64	17.9	72	28.6	
Other	137	21.0	102	25.4	35	13.9	
**Duration of stay in community (years)**							<0.001
0-< 1	164	25.1	57	14.2	107	42.5	
>1–5	289	44.2	201	50.0	88	34.9	
> 5	201	30.7	144	35.8	57	22.6	
**Away from home ≥ 2 nights, last 3 month**	299	45.8	221	55.1	78	31.0	<0.001
**Number of sexual partners, last 3 month**							<0.001
1	215	32.8	80	19.9	135	53.6	
2 to 3	333	60.0	230	57.2	103	40.9	
4 or more	106	16.2	92	22.9	14	5.6	
**New sexual partner, last 3 month**	480	74.1	324	81.2	156	62.7	<0.001
**Condom use with new partner, last 3 month**	305	63.5	205	63.3	100	64.1	0.859
**Drank alcohol, in last month**							0.003
Never	239	36.6	128	31.9	112	44.1	
Sometimes	354	54.1	229	57.0	126	49.6	
Daily	61	9.3	45	11.1	16	6.3	
**Drank alcohol before sex, in last month**							0.005
Never	352	53.8	197	49.0	155	61.5	
Sometimes	204	31.2	135	33.6	69	27.4	
Always	98	15	70	17.4	28	11.1	
**Drug use, in last month**							<0.001
None	570	87.2	334	83.1	236	93.7	
Marijuana	35	5.3	25	6.2	10	4.0	
Khat	41	6.3	39	9.7	2	0.8	
Other	8	1.2	4	1.0	4	1.6	
**Genital sores, in last 3 months**	272	41.6	145	36.1	127	50.4	<0.001
**Laboratory confirmed syphilis**	23	3.5	16	4.0	7	2.8	0.417
**Circumcised**	—	—	164	42.0	—	—	

^1^Drying fish, salting or smoking fish

Compared to men, women were significantly more likely to be older, have more than primary education, and report having only one sexual partner in the previous month. Men were more likely to be single, divorced or widowed, engaged in fishing related occupations, have lived in the community for five years or more, spend more nights away from home, and always drink alcohol before sex.

### Study drop out

Among the 654 participants, 197 (30.1%) dropped out of the study (Table 2), including the 72 who did not return for the first three-month follow-up visit. The overall dropout rate was 25.3 / 100 PYO (95% CI: 22.0–29.1). If the 72 volunteers who never returned are not included in the sample, the dropout rate for the remaining 582 participants was 17.1/100 PYO (95% CI 14.4–20.4). The most common reasons for dropping out among the 197 participants who didn’t complete the study included moving away from study area (56.9%) withdrawal from the study (15.7%), death (1.5%), and not being traceable so that the reason for drop-out was unknown (13.2%). Fifteen participants were censored at the 12-month visit because they no longer qualified as being at high risk of HIV.

**Table 2:** Demographic and behavioural factors associated with study drop out (DO).

Variable	N	DO	PYO	DO/100PYO	RR(95% CI)	LRT P-value	aRR(95% CI)	LRT p-value
**Overall 2 year drop out**	654	197	778.9	25.3				
**Gender**								
Male	402	103	507.9	20.3	Ref	<0.001	Ref	0.002
Female	252	94	270.9	34.7	1.7(1.29–2.26)		1.56(1.12–2.18)	
**Age (years)**								
35+	124	25	166.3	15	Ref	0.004	Ref	0.007
25–34	282	88	340.3	25.9	1.72(1.10–2.68)		1.63(1.04–2.55)	
18–24	248	84	272.2	30.9	2.05(1.31–3.21)		1.64(1.03–2.60)	
**Current marital status**								
Single	186	64	217.4	29.4	Ref	0.107		
Married	319	85	394.5	21.5	0.73(0.58–1.01)			
Separated/Widowed	149	48	166.9	28.8	0.98(0.67–1.42)			
**Educational level**								
More than primary	125	39	156.0	25	Ref	0.013	Ref	0.01
Primary	466	131	563.3	23.3	0.93(0.65–1.33)		1.06(0.74–1.52)	
None	63	27	59.5	45.4	1.82(1.11–2.97)		2.02(1.23–3.31)	
**Occupation**								
Small scale business	49	8	68.3	11.7	Ref	0.006	Ref	0.035
Fishing/fishing related^1^	334	99	413.2	24	2(0.99–4.21)		1.98(0.95–4.11)	
Services (Bar/lodge/restaurant/saloon)	67	17	80.0	21.2	1.8(0.78–4.21)		1.18(0.50–2.78)	
Other	204	73	217.3	33.6	2.9(1.38–5.95)		2.05(0.98–4.31)	
**Religion**								
Muslim	151	41	187.8	21.8	Ref	0.272		
Christian	503	156	591.1	26.4	1.21(0.86–1.70)			
**Tribe/Ethnic group**								
Baganda	288	83	339.2	24.5	Ref	0.762		
Banyankole	93	29	103.1	28.1	1.15(0.75–1.75)			
Banyarwanda	136	39	170.3	23	0.94(0.64–1.37)			
Other	137	46	166.3	27.7	1.13(0.79–1.62)			
**Duration of stay in community (years)**								
>5	201	41	277.8	14.8	Ref	<0.001	Ref	0.004
1 to 5	289	92	341.3	27	1.83(1.26–2.64)		1.68(1.16–2.45)	
0-<1	164	64	160.0	40	2.71(1.83–4.02)		2.22(1.46–3.38)	
**Away from home ≥ 2 nights, last 3 months**								
No	354	107	425.1	25.2	Ref	0.912		
Yes	299	90	351.9	25.6	1.02(0.77–1.34)			
**Number of sexual partners, last month**								
0–1	215	64	261.7	24.5	Ref	0.565		
2 to 3	333	97	396.8	24.4	0.99(0.73–1.37)			
4 +	106	36	120.4	29.9	1.22(0.81–1.84)			
**New sexual partner, last 3 month**								
No	168	48	205.9	23.3	Ref	0469		
Yes	480	148	563.7	26.3	1.12(0.81–1.56)			
**Condom with new partner, last 3 month**								
No	175	60	198.7	30.2	Ref	0.322		
Yes	305	88	365.0	24.1	0.79(0.57–1.11)			
**Drank alcohol, last month**								
Never	293	80	280.5	22.9	Ref	041		
Sometimes	354	98	418.9	19.2	0.82(0.61–1.10)			
Daily	61	19	79.4	15.3	0.84(0.51–1.38)			
**Drank alcohol before sex, last month**								
Never	239	113	407.8	27.7	Ref	0.368		
Sometimes	354	58	254.3	22.8	0.82(0.59–1.13)			
Always	61	26	116.8	22.3	0.8(0.52–1.23)			
**Illicit drugs use, in last month**								
None	570	169	680.5	24.8	Ref	0.754		
Khat	35	11	46.8	23.5	0.94(0.51–1.74)			
Marijuana	41	14	42.8	32.7	1.32(0.76–2.27)			
Other	8	3	8.9	33.7	1.35(0.43–4.24)			
**Genital sores, in last 3 months**								
No	382	114	454.7	25.1	Ref	0.884		
Yes	272	83	324.1	25.6	1.02(0.77–1.36)			
**Laboratory Confirmed Syphilis**								
No	591	180	695.1	25.9	Ref			
Yes	63	17	83.7	20.3	0.78(0.48–1.36)			
**Male circumcision**								
Yes	164	64	296.3	21.6	Ref			
No	231	37	202.6	18.3	0.84(0.56–1.27)			

^1^Drying fish, salting or smoking fish

PYO (Person years of observation)

Over two years, 45 participants became HIV-infected. The HIV incidence was 6.04 p/100 PYO (95% CI: 4.36–8.37), and has been reported previously [15].

Factors that remained independently associated with dropping out in the adjusted analysis included being female (adjusted rate ratio [aRR] = 1.56; 95% CI 1.12–2.18), having no education (aRR = 2.02; 95% CI: 1.23–3.31) being 18–24 years old (aRR = 1.64; 95% CI: 1.03–2.06) or 25–34 years old (aRR = 1.63; 95% CI: 1.04–2.55) compared to being 35 or older. Participants who had lived in the fishing community for one year or less were two times more likely to drop out (aRR = 2.22; 95% CI: 1.46–3.38) compared to those who had spent over 5 years.

When we compared the 582 who completed at least one follow up visit, to the 72 who never returned for any visit, those who never returned were: younger (mean age of 25.5 vs. 28.0; p = 0.004), more likely to have had no education (19.4% vs 8.4%, p = 0.010) and to have lived for less than a year in the community (45.8% vs 22.5%, p<0.001).

## Discussion

**Fig 1:**
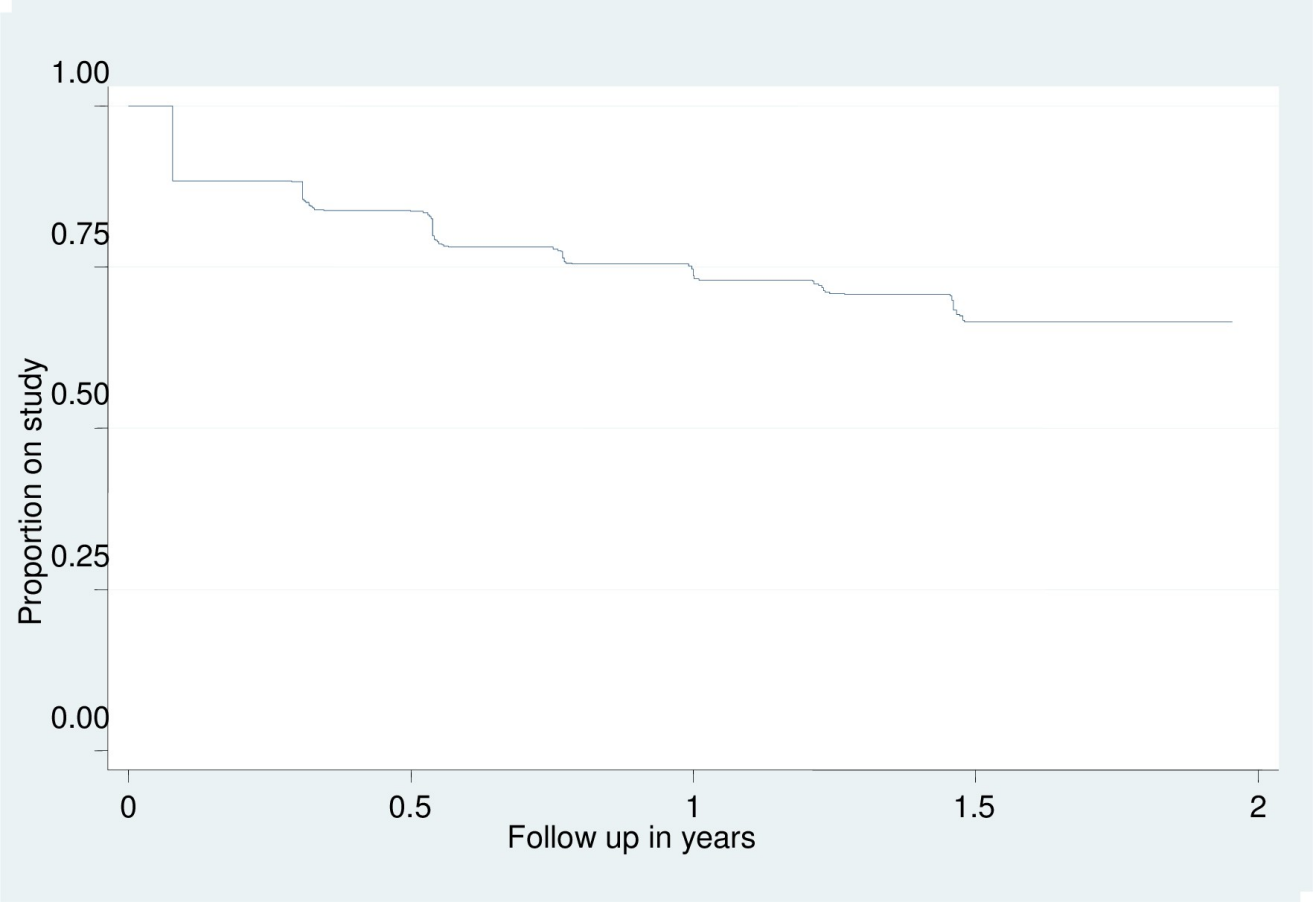
Kaplan Meier plot of volunteer attrition over time.

In this HIV vaccine preparedness study among individuals from fishing communities, we observed a dropout rate of about 25% per year. These findings show similar levels of dropout as reported from cohorts recruited from other fishing communities around Lake Victoria, with annual retention rates of 85% [11] and 77% [12]. Our study differs from others, however, as our research site was distant from the participating communities, and we also evaluated retention over a 2-year period. We found that a significant proportion (23.5%) of participants did not return for any followup visits, or only came for the first three-month visit Fig 1. This suggests that a lead-in enrolment scheme may be needed to recruit participants from these communities for actual clinical trials.

Even though we provided reimbursement for transportation and time, as well as free STI treatment, this may not have been adequate to overcome the hurdles of having to travel to a distant research site. In a study conducted among discordant couples in Kenya, participants who were living 5–10 kilometres away from the treatment clinic were twice as likely to have follow-up interruptions compared to those living less than 5 kilometres away [16]. Although no differences were observed in dropout rates between the four different fishing communities, it is possible that the distance from the fishing communities to the clinic could have contributed to the drop out.

We found that persons who had lived in the community for a relatively short time were more likely to drop out of the study, and is consistent with other similar studies [11, 12]. Living in a community for a shorter period of time may be a proxy for people who are more likely to be migratory and/or to move between different islands and landing sites following the seasonal fishing seasons [17].

We also found that retention among women was lower than among men. This may be because women’s roles in the family are time-demanding, such as caring for sick family members and children. It is also possible that some women enrolled in our study were engaged in transactional sex, and might move from place to place based on the demand for their services; however, we did not collect specific information about commercial sex work. Since it is important to ensure that men and women are equally engaged in HIV vaccine efficacy trials, efforts need to be made to increase female enrolment and retention in clinical trials within these communities. Participation of women from the fishing communities can potentially be improved either by involving individuals or groups in the community who have ample influence on them, such as, their spouses, brothel owners, bar and lodge owners in research activities or increasing reimbursement, so that it is financially helpful.

We note that younger participants are more likely to drop out a finding similar to what was observed in the previous studies [11, 12] and may be due to the fact that young people possibly haven’t settled in any stable relationship/occupation and are still moving in search of more economically rewarding livelihoods.

Our study had some limitations. Firstly, we did not obtain information of the specific reasons why participants dropped out. We also do not have data on whether women were breastfeeding or pregnant, which may have influenced their ability to return for followup. Secondly we are not able to estimate what proportion of people reached through community meetings or who were HIV tested, who subsequently decided to enrol. This information would allow us to evaluate the effectiveness of our recruitment procedures. Finally, because we removed people at the annual visit who were no longer eligible, this will have reduced retention estimates, although the number who were withdrawn was small. However, vaccine and other HIV prevention trials require participants to be at high risk, so removing these people from the study, was in keeping with procedures that would be implemented in an actual trial.

## Conclusions

Although we demonstrate ability to enrol participants in this longitudinal cohort, the high dropout rate especially in the first three months is a point of concern. Inclusion of volunteers into clinical trials to evaluate products such as HIV vaccine candidates would require delayed enrolment and consider an initial pre-screening period of approximately 2–3 months, requiring potential volunteers to attend more than one screening visit to establish commitment. Particular attention for retention should be paid to the participant categories identified as being a risk of dropping out such as female, younger, lower education and participants with short duration of stay in the community.
